# ‘Smiles and laughter and all those really great things’: Nurses' perceptions of good experiences of care for inpatient children and young people with intellectual disability

**DOI:** 10.1111/jan.15256

**Published:** 2022-04-22

**Authors:** Laurel Mimmo, Michael Hodgins, Nora Samir, Joanne Travaglia, Susan Woolfenden, Reema Harrison

**Affiliations:** ^1^ Clinical Governance Unit The Sydney Children's Hospitals Network Sydney NSW Australia; ^2^ Population Child Health Research Group School of Women's and Children's Health Faculty of Medicine University of New South Wales Sydney NSW Australia; ^3^ Centre for Health Services Management Faculty of Health University of Technology Sydney Sydney NSW Australia; ^4^ Centre for Health Systems and Safety Research Australian Institute of Health Innovation Faculty of Medicine, Health and Human Sciences Macquarie University Sydney NSW Australia

**Keywords:** brilliant care, child life therapy, children, healthcare quality, hospital, intellectual disability, nurses/midwives/nursing, Paediatrics, patient experience, young people

## Abstract

**Aim:**

To understand what constitutes a good experience of care for inpatient children and young people with intellectual disability as perceived by nursing staff.

**Design:**

Interpretive qualitative study.

**Methods:**

Focus groups with clinical nursing staff from speciality neurological/neurosurgical and adolescent medicine wards across two specialist tertiary children's hospitals in Australia were conducted between March and May 2021. Data analysis followed interpretative analysis methods to develop themes and codes which were mapped to a conceptual model of safe care.

**Results:**

Six focus groups with 29 nurses of varying experience levels were conducted over 3 months. Themes and codes were mapped to the six themes of the conceptual model: use rapport, know the child, negotiate roles, shared learning, build trust and relationships, and past experiences. The analysis revealed two new themes that extended the conceptual model to include; the unique role of a paediatric nurse, and joy and job satisfaction, with a third contextual theme, impacts of COVID‐19 pandemic restrictions. With the perspectives of paediatric nurses incorporated into the model we have enhanced our model of safe care specifically for inpatient paediatric nursing care of children and young people with intellectual disability.

**Conclusion:**

Including perceptions of paediatric nurses confirmed the position of the child with intellectual disability being at the centre of safe care, where care is delivered as a partnership between nursing staff, child or young person and their parents/family and the hospital systems and processes.

**Impact:**

The enhanced model offers a specialized framework for clinical staff and health managers to optimize the delivery of safe care for children and young people with intellectual disability in hospital.

## 1 INTRODUCTION

Children and young people (CYP) with intellectual disability have higher incidence of medical conditions and associated healthcare needs than their non‐disabled peers, often necessitating inpatient admissions and stays in hospital of longer length (Einfeld et al., 2011; Iacono et al., 2014; Mimmo et al., 2021; Oeseburg et al., 2011). The limited published studies on the inpatient experiences for CYP with intellectual disability, or their parent/carer, have consistently reported healthcare staff reliance on parental presence and inability to identify the needs of the child, resulting in confusion about roles, ineffective partnerships in care and poor hospital experiences (Mimmo et al., 2018; Mimmo et al., 2019). Attempts to address poor healthcare experiences for inpatient CYP with intellectual disability have largely focussed on challenges and barriers for healthcare workers in the delivery of high quality and safe care (Aston et al., 2014a; Oulton et al., 2018).

Understanding challenges and barriers in service delivery is important for health services to identify safety deficits and areas for improvement in care delivery, but has provided limited success in creating healthcare improvement due to health system complexity (Leape et al., 2009). Unlike mechanical systems, the component parts of a health care system are characterized by diversity, interconnectedness, inter‐dependency and adaptation to input; they do not act in a linear way with predictable outcomes. (Hollnagel et al., 2015). A reorientation of analyses of experience towards a strengths‐based approach that explores positive experiences is therefore increasingly promoted through concepts of ‘positive deviance’ and ‘brilliant care’ that is, those experiences of care that go beyond expectations (Baxter et al., 2016; Dadich et al., 2015). Our study reflects this paradigm shift in contemporary healthcare improvement research by investigating and understanding the actions and processes involved when things go right promoting learning from the presence of safety (Hollnagel et al., 2015). Drawing on these approaches, our study sought to explore paediatric nurse perceptions of good hospital care experiences for CYP with intellectual disability from nurses who work on wards that admit these children on a regular basis.

## 2 BACKGROUND

Approximately 1%–3% of the global population has intellectual disability (Maulik et al., 2011). In Australia, 4.5% of children aged 15 years or under have intellectual disability (Australian Bureau of Statistics, 2018). A recent study found almost 14% of admissions to a tertiary paediatric healthcare organization were CYP with intellectual disability (Mimmo et al., 2021). These CYP also had more frequent admissions, a significantly longer median length of stay, and higher median cost of admission than their peers (Mimmo et al., 2021). At the same time, inpatient CYP with intellectual disability and their parents consistently report poor quality and safety experiences. These related to healthcare staff assumptions and lack of knowledge regarding the child with intellectual disability and their needs and preferences, reliance on parental presence and not listening to parental expertise, all of which were perceived to inhibit healthcare staff's ability to plan and adapt care delivery to the needs of the child (Mimmo et al., 2018; Mimmo et al., 2019; Oulton et al., 2018). Despite these inequities in quality and safety outcomes, increased healthcare utilization, and poor experiences of care, there remains a paucity of targeted research to explore and understand nursing insights into positive inpatient experiences of healthcare for CYP with intellectual disability using a quality and safety lens.

In inpatient settings nurses and nursing teams are the healthcare professional group most consistently in contact with patients; nurses are accessible to patient bedsides 24 h a day, 7 days a week, and are responsible for the co‐ordination of day to day inpatient care delivery. Nurses and midwives account for almost half the global health workforce (World Health Organisation, 2020); in the Australian and English public health systems, nurses and midwives account for over half the clinical workforce (Australian Institute of Health and Welfare, 2020; The King's Fund, 2020), and in the United States (US) comprise at least one third of hospital employees (U.S. Department of Labor, 2020). Because of this, measures of quality and safety, and patient experiences of care are inherently biased to patient perceptions of the quality of nursing care (Edvardsson et al., 2017). Nurses report fear of caring for people with intellectual disability, identifying lack of knowledge, skills, experience and exposure to patients with intellectual disability as key factors of these negative perceptions consistently described across the international literature (Appelgren et al., 2018; Iacono et al., 2014). The few studies to date regarding the nurses' experiences of caring for CYP with intellectual disability in hospital settings report challenges with identifying and acting on the needs of the child, lack of specific education, finding time to provide care and the importance of the parent‐nurse relationship (Aston et al., 2014b; Lewis et al., 2019; Oulton et al., 2018). Missing from the literature are studies exploring the perceptions and experiences of good quality care for inpatient CYP with intellectual disability from nurses who routinely care for these children, and contributions to evidence of what constitutes good experiences of hospital for this marginalized group of children.

### 2.1 Conceptual model

**FIGURE 1:**
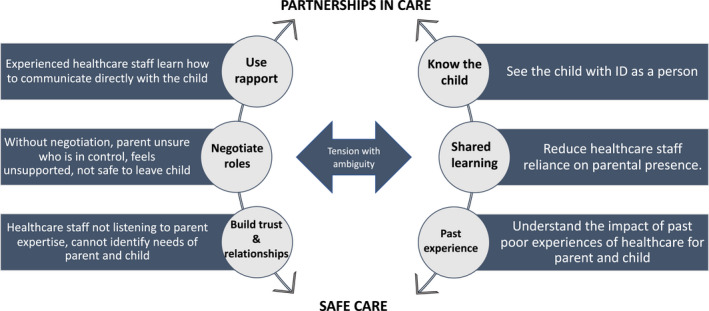
Conceptual model of safe care for a child with intellectual disability in hospital

This study is part of a broader body of work comprising the lead author's doctoral studies, exploring quality and safety outcomes, and patient experience, for inpatient CYP with intellectual disability. Preceding this current study was the development of a conceptual model of safe care (see Figure 1) arising from a metanarrative of experiences of parents with a child with intellectual disability in hospital (Mimmo et al., 2019).

Themes from the model in Figure 1 suggest positive experiences of care for parents of children with intellectual disability were underpinned by partnerships of care with healthcare staff. Central to these partnerships were staff using rapport to get to know the child with intellectual disability; negotiating roles and shared learning by listening to parental expertise to understand the child's unique care needs in hospital; building trust and relationships; and appreciating that negative healthcare experiences can impact on expectations for future healthcare experiences (Mimmo et al., 2019). For this study, we wanted to build on this conceptual model and understand how the model may be practically applied in the care of CYP with intellectual disability in an acute paediatric healthcare setting. To do this we sought the perspectives of paediatric nurses at the frontline of inpatient care delivery, focussing on good experiences of hospital care for a CYP with intellectual disability.

## 3 THE STUDY

### 3.1 Aim

The aim of this study was to understand what constitutes a good experience of care for inpatient CYP with intellectual disability as perceived by nursing staff. Our research question was, how do nurses conceptualize a good hospital experience for inpatient CYP with intellectual disability? The perceptions of nurses were then mapped to perceptions of parents to enhance our conceptual model of safe care (Mimmo et al., 2019).

### 3.2 Design

An interpretive qualitative study design was used for this study (Sandelowski, 2000). Using qualitative inquiry to derive evidence of the ‘how to’ from those who have expertise in the practice of the healthcare delivery under study, in this case paediatric nurses who routinely care for CYP with intellectual disability, will give clinical and practical relevance to the findings (Leeman & Sandelowski, 2012).

### 3.3 Sample/participants

Clinical nursing staff from two speciality neurological/neurosurgical and two speciality adolescent medicine wards across the two specialist tertiary children's hospitals of a single paediatric healthcare organization in Australia were invited to participate in the focus groups. Our purposeful recruitment strategy in these specialist wards sought to explore and capture data from nurses who routinely delivered inpatient care to CYP with intellectual disability, and would therefore likely have many instances upon which to draw.

### 3.4 Data collection

#### 3.4.1 Method

Focus groups were conducted to explore perceptions of good care experiences for inpatient CYP with intellectual disability, asking paediatric nursing staff to reflect on their experiences of providing good care and determine what constitutes good experiences of hospital care for this group of children. The use of focus groups encourages participants to bounce ideas and thoughts off each other and can support others to contribute thoughts on their shared experiences (Pope et al., 2002) which may otherwise not have occurred during a one on one interview. The power of focus groups is through fostering co‐created meaning and ‘collective sense‐making’ as participants discuss their experiences between themselves and the facilitator (Wilkinson, 1998).

The perceptions of the nursing staff were mapped to the conceptual model of safe care, following the Framework method described below (Gale et al., 2013). This was to inform enhancements to our conceptual model of care and future clinical education programs regarding improving care quality in the hospital setting.

#### 3.4.2 Procedure

Focus group dates and times were pre‐arranged with each ward nursing unit manager (NUM) and ward based clinical nurse educator (CNE) to minimize conflicts with clinical duties, enabling attendance for those with a mainly clinical workload. In consultation with each ward NUM and CNE the optimum approach agreed was to conduct the focus groups during the ward's routine in‐service time. Where necessary to comply with Public Health orders regarding public gatherings the facilitator conducted the focus groups using an online videoconference platform, Zoom™.

At the beginning of each group the lead author explained the purpose of the focus groups, introduced the facilitator, and ground rules were established. Prior to commencing the focus groups the purpose and topic guide was discussed between the lead author and the two facilitators, who are health services researchers but not clinicians. Additional prompts and probes were added to reflect some nuances of the health service. The first focus group was conducted by one facilitator with the other as an observer for consistency in how the groups would run. The participants were asked to reflect on their experiences of providing inpatient nursing care to CYP with intellectual disability, and what they perceived to be good experiences for these CYP. The topic guide template used across the focus groups is attached as supplementary file one (Appendix A).

### 3.5 Ethical considerations

Ethical approval for this study was obtained from the organization's Human Research Ethics Committee, reference number: 2019/ETH13465. Support from the site based Directors of Nursing and NUMs was sought for recruitment of staff participants, including distributing information sheets during department meetings and via department emails; paper copies were available in the NUM's office a minimum of 1 week before each scheduled focus group. Written consent was obtained from all participants at the time and prior to commencement of each focus group. To maintain anonymity of focus group participants the audio recordings were sent directly for transcription by a professional service. These transcripts were then used for analysis.

### 3.6 Data analysis

Our analysis was inductive and iterative, and undertaken over two phases. In the first phase the data analysis followed the analytical steps recommended for Interpretive Phenomenological Analysis (IPA) methodology, ideal for sense‐making of lived experience data (Smith et al., 2008). Detailed and iterative analyses of transcripts are done on a case by case basis, hence small samples are recommended (Smith & Osborn, 2003). The lead author engaged in immersion of transcripts of the focus groups through repeated reading and then writing of initial codes and themes for sense‐making of the participants' stories of good experiences of care, and conducted initial interpretive analysis of the transcript of the first focus group.

One or two focus groups were conducted with each ward, with the option of further groups if new concepts and themes emerged for further exploration. However, after six focus groups were conducted across the four wards it was agreed between the lead author and the two focus group facilitators that data saturation had been achieved through the collection of both thick and rich data with consistent themes across focus groups (Fusch & Ness, 2015). Shortly after the final focus group the two facilitators reviewed the transcripts independently and met with the lead author to review the initial codes and themes developed. The remaining authors read the transcripts independently and then met with the lead author to review and agree on the codes and themes. The codes and themes were then discussed at length between the lead author and focus group facilitators before being reviewed and refined further through discussions with the broader authorship team. Using IPA we were able generate the initial codes and themes that encapsulated the unique perspective of the nurses.

**FIGURE 2:**
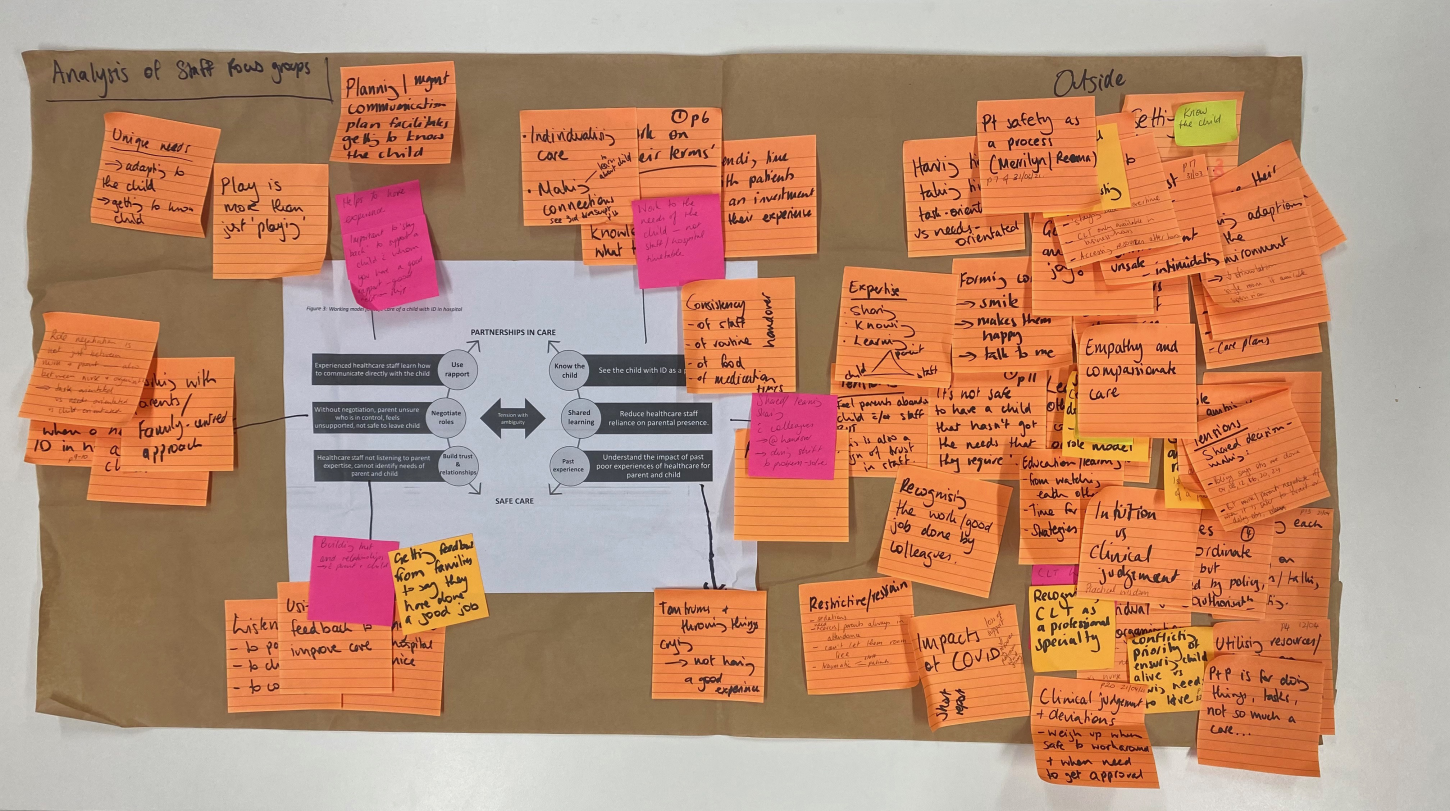
Mapping themes to conceptual model

In the second phase we applied the Framework Method, described by Gale et al. (2013), to map the codes and themes to the current conceptual model and identify novel themes. The six components of the conceptual model made up the Framework rows; each focus group was classified as a case and codes applied within and across the cases (Gale et al., 2013). The codes and themes were grouped into those that aligned with the conceptual model and themes that were significant but sat outside the conceptual model, classified as orphan themes. This process was then followed for the subsequent focus groups, with each transcript considered on its own, then against the other focus groups, as per IPA methodology. The codes were then themed and mapped according our conceptual model of safe care (Figure 1), branching out from each arm, see Figure 2.

### 3.7 Validity and reliability/rigour

In discussing validity and reliability/rigour, we draw on the work of Mays and Pope (1995) and Morse's critique of determining rigour in qualitative studies (Mays & Pope, 1995; Morse, 2015). Rigour was ensured by seeking thick description, awareness of bias and closeness to the data, peer review and reflexive practice. Thick description was achieved through our participant sampling strategy and use of focus groups with specific wards that routinely care for CYP with intellectual disability within the same healthcare organization.

#### 3.7.1 Reflexivity

As the lead author is a registered nurse employed within the clinical governance unit of the healthcare organization the focus groups were facilitated by university research staff with contingent staff appointments with the healthcare organization. Appreciating the lead author is close to the data and research topic, from a professional and research perspective, it was important to both acknowledge this bias while leveraging the lead author's common standing with the participants to establish trust. Peer review and debriefing between the research team was conducted throughout the analysis and write up of the findings. A summary of the findings has been shared with the ward NUMs and nurses who participated and requested a summary, along with the organization's nursing executive team.

Pillow (2003) notes reflexive practice ‘as critical to exposing the difficult and often uncomfortable task of leaving what is unfamiliar, unfamiliar’ (Pillow, 2003, p. 177). Researchers decide what is included in the description (Sandelowski, 2000); applying reflexivity to do this is challenging, it requires a fine balance (Pillow, 2003). The lead author is an experienced paediatric nurse with clinical and research interest and skill caring for CYP with intellectual disability, who had, at the time of the study, been working for the organization for almost 12 years. To mitigate the potential impact of the lead author's familiarity with the participants, their practice and clinical context on the frankness of focus group discussions the lead author was not present while the focus group discussions took place. In addition, the focus group facilitators emphasized that the aim was to share and celebrate their experiences of good care delivery. To promote reflexivity the lead author maintained personal notes during the period of analysis, noting the thoughts and reflections that arose when reading the transcripts, and discussed these at length with the authorship team.

## 4 FINDINGS

A total of six focus groups were conducted between March and May 2021 with 29 nursing staff of varying seniority and experience from the four speciality wards. Duration of focus groups varied from 26 to 43 min. Nursing staff participating included Endorsed Enrolled Nurses (1), Registered Nurses (18), new graduate/Transitioning Registered Nurses (1), Nursing Unit Managers (1), Clinical Nurse Educators (4), Clinical Nurse Specialists (1) and university nursing students (3). Focus group sizes ranged from two to ten participants and we did not collect details on age, gender or years of experience.

Our findings are presented against the six components of the conceptual model (see Figure 1); use rapport, know the child, negotiate roles, shared learning, build trust and relationships, and past experiences. For each component theme we have included a table of codes and key extracts from the focus groups as they relate to each theme.

In addition, we identified three new themes. Two of these themes extend and enhance the model; the unique role of a paediatric nurse, and joy and job satisfaction. The third theme reflects the context when the focus groups were conducted; impacts of COVID‐19 pandemic restrictions. These themes are presented after the conceptual model themes with a table summary of key extracts related to these new themes. We then present our enhanced model of safe care for a child with intellectual disability in hospital.

### 4.1 Use rapport

In all the focus groups the participants agreed that using rapport to build familiarity with a CYP with intellectual disability helped the child feel safe and comfortable in hospital. The groups identified the importance for nurses to use play and rapport to form connections with CYP where they had had some distressing experiences. Some of the discussion suggests the nurses felt these connections supported a CYP with intellectual disability in coping with challenges during hospitalization. It was clear that these connections also brought joy to the whole nursing team, not just the nurses directly involved in a child's care.

While specific policies and procedures were not identified for caring for a child with intellectual disability, one group suggested policies may help guide inexperienced nurses to involve parents and use play for rapport to get to know a child with intellectual disability. Key focus group extracts related to this theme are presented in Table 1.

**TABLE 1:** Use rapport; key extracts from focus groups

Use rapport	Key extracts from focus groups	Focus group reference
Form connections through play	*Participant: […] we had one patient who was extremely difficult with a medical prognosis as well as an intellectual disability, and this child was needing a lot of extra care, […]. It was really lovely to be able to see him and us interact when things were going good for him. […] when we were having great days, it was nice to be able to interact with him*. *They would be singing songs, and we'd put videos on that he would enjoy, […] Listening to him laughing […]. I remember the first time I ever heard him laughing, and it was just such a joy to be able to hear him laugh […] It was a really sweet thing*.	FG4

	*Participant: […] use your interpersonal skills and rapport building, […] make the young person feel comfortable and feel relaxed. […] can really make a big difference in their comfort levels and hospital experience*.	FG5
Policies	*Participant: […] making sure that you are involving the parents, involving—like family centred care. […]*	FG3
	*Participant: […] for new staff or inexperienced staff things like that might be useful […]*.	

### 4.2 Know the child

The nurses agreed that knowing a CYP had intellectual disability, especially before arriving on the ward, allowed them to prepare and adapt their care delivery to suit the CYP's needs. The nurses described ways in which they used knowledge of a CYP with intellectual disability to facilitate care planning, arrange for specialized equipment, and make adaptions to the ward environment to optimize safety and quality.

The nurses identified that not being able to get to know a child because there was no carer was problematic, particularly if a child was in discomfort, and the nurses sought other ways to find out about the child. The nurses also recognized the importance of knowing the needs of a CYP with intellectual disability to optimize their care, in particular their method of communication, and used various strategies to capture key pieces of information from parents/carers.

Across the focus groups getting to know the CYP with intellectual disability recognized their innate humanity. They felt it was important to treat a CYP with intellectual disability as a human being; preserving dignity and advocating for the child, particularly when managing pain and end of life, and using empathy and compassion. Key focus group extracts related to this theme are presented in Table 2.

**TABLE 2:** Know the child, key extracts from focus groups

Know the child	Key extracts from focus groups	Focus group reference
Prepare and adapt before admission	*Participant:… for patients that are coming in for a booked admission, […] our CNSs* [clinical nurse specialists] *will have communicated with them […]. they'll get a bit of a history on the child […] they'll find out about the intellectual disability […] if they then do need to be in a quieter room or, […] if they are an absconding risk*. *[…] do we need to put things in place to make this a safer environment for them […]*.	FG2
	*Participant: […] a lot of our kids, they'll have introduction booklet that the parents prepared for [….] as an overview of what they like or what they do not like and what to look out for*.	FG6
Know what the child needs and find ways to communicate	*Participant: One thing I found a little distressing […] where they come in and there's no support person, there's no carer, and they are very disabled physically, […] and you do not know how to make them comfortable and no one's there to tell you how[…]*.	FG4
	*Participant: I think it is really important to actually spend time having a chat with the parents and potentially the child about how they best communicate*.	FG5
Preserving dignity, using empathy and compassion	*Participant: […] talking to the patients whether or not they can talk back to me […] treating them as though they can listen so talk to them […] just say, hi, […] treat them with dignity*.	FG3
	*Participant: Sometimes with our palliative kids, we'll get really creative and we'll put fairy lights in their room, or we'll paint their nails*.	FG4

### 4.3 Negotiate roles

Nurses described negotiations with parents to come to mutual agreement on the care needs of their child as part of their family and child centric approaches while others espouse that the prevailing practice in the unit was the family centric model. For some nurses having a parent or carer at the bedside to ‘help us look after them’ was how they practiced family centred care, and seen as a marker for ensuring a good experience for a child with intellectual disability. The parent/carer was available to tell them what their child needed and so they could help attend to the child's daily care needs. For some nurses not having a parent or carer at the bedside was considered ‘bad’ as it ‘ruins everything’; others recognized their role in supporting parents, as there may be additional or conflicting responsibilities for parents to manage on top of their hospitalized child.

Descriptions of role conflict arose when nurses described trying to negotiate a child's care needs against the needs and capabilities of the hospital/organization. Senior nurses described how, as they gained experience, they would use their clinical judgement to negotiate and prioritize the care needs of a CYP with intellectual disability against their clinical responsibilities. All nurses viewed the time constraints on their practice and hospital routines as contributors to poor care quality experiences for CYP with intellectual disability, and identified how this may also worsen a child's medical condition and cause unnecessary stress.

As above, nurses reported conflict in their role to provide the attention and time the child needs, such as play and company, versus the clinical requirements of their role, resulting in triaging clinical need ahead of the child's needs. The role of play and play specialists, called Child Life Therapists in this context, viewed as being just as important as other health professionals in the care of CYP with intellectual disability, was raised in every focus group. Key focus group extracts related to this theme are presented in Table 3.

**TABLE 3:** Negotiate roles; key extracts from focus groups

Negotiate roles	Key extracts from focus groups	Focus group reference
Delivering family centred care	*Participant: It [family centred care] is incorporating the family in decision‐making and getting to know the parents and/or the carers and the child and the particular triggers and the things that they are afraid of, for instance. Or the things that they really enjoy doing […]*.	FG2
	*Participant: […] each child with an intellectual disability is going to have communication challenges unique to them […] their families are going to be the best way to know whether they are in pain, they are upset, if they are happy, whatever's going on*.	FG3
Helps to have a carer at the bedside	*Participant: I think it's bad [when a child with intellectual disability does not have a carer with them] because we often aren't really familiar with the child. We also cannot provide them that one on one care that they really need, because we do not have the staffing for that. […] these kids are super complex. […] we usually practise like what—family‐centred care here, we cannot do that with a child, because it's just them. […] hard for them, and that's hard for us*.	FG4
Negotiating time and role conflicts for nurses with meeting needs of CYP with needs of organization	*Participant: […] there's a lot of disruption to their routine which for a lot of kids is really upsetting. […it] often makes them worse or triggers more seizures […] it's quite distressing for a lot of patients and carers*.	FG3
	*Participant: […] it's my job. […] if a parent or carer is not there, spending that individualized time with that child. […] nurses now, you are a lot more time poor, so you do not necessarily have that time to allocate, to sit down and go, let us draw a picture about how you are feeling, or let us draw a picture about how you best communicate*.	FG5
The role of play and child life therapy	*Participant: Yeah. I've seen—I would say there's been like adultification of the services a little bit […]. More focused on clinical things not how a child responds or what a child needs. Play, the fun…* *[…]* *Participant: […] people say, oh, nursing staff can do this and this and more and more and more things but the more things you add to it, including child—Child Life Therapy, the lower the quality is going to get. Nursing staff are going to triage things that need to be done […]. There's only so many hours in a day and you need to be able to do things that are critical for the child's life before things that the child should have in their life*.	FG3

### 4.4 Shared learning

Nurses readily identified that effective communication with parents and carers was critical for optimizing care quality and safety during the admission and for a good care experience. Parents and carers were sought out by the nurses both for guidance on and learning about the care needs of a CYP with intellectual disability. If a parent or carer was not available at the bedside the nurses looked for other means such as communication folders or bedside communication boards to learn about the needs of the CYP. In particular, learning how the CYP with intellectual disability communicated was a key facet for ensuring good experiences of care.

Through the focus group discussions communication across the multidisciplinary team was identified as an important channel for shared learning. This was evident in discussions on how colleagues in other teams or disciplines shared their knowledge of a CYP with intellectual disability with ward staff in planning for and realizing a successful admission. In the same vein, poor communication between teams despite extensive planning can have negative flow on effects, especially with regard to discharge planning.

The nurses valued learning from each other and sharing knowledge learnt from the parent or their child with other colleagues, not just other nurses. Some identified that shared learning gave opportunity to act as advocates for their patients, or to support new staff, such as new graduate nurses and new doctors, who may be unfamiliar with the specific needs of the CYP with intellectual disability.

Learning about good care by observing each other was considered particularly important for less experienced nurses in the focus groups. A senior nurse revealed at the end of one group that some discussion initiated by a junior nurse helped them to realize that they are role modelling good care, that less experienced staff watch how senior nurses interact with CYP with intellectual disability, and then mimic these techniques and strategies to improve their own practice.

Transitions of care and handover were repeatedly discussed as important touch points for the shared learning and consistency that reflected good experiences for CYP with intellectual disability. The nurses reported needing to make sure their patient continued to received good care after they had finished their shift. This was particularly important when caring for a CYP with intellectual disability, as handing over all the necessary information to their colleagues often resulted in staying late or over time, which the nurses perceived was unrecognized by management as part of delivering good quality care. Key focus group extracts related to this theme are presented in Table 4.

**TABLE 4:** Shared learning; key extracts from focus groups

Shared learning	Key extracts from focus groups	Focus group reference
Through effective communication	*Participant: We sort of get guided by [a parent], so they have input as well, as how to talk to the child […]*.	FG2
	*Participant: […] that goes back to having a chat with the parents as well, we are going to do this procedure, how do you feel your child will be best prepared? […] having a good conversation and explaining the procedure, […] what's going to work best for that child*.	FG5
Good communication and discharge planning have positive flow on effects	*Participant: If there is good communication from the beginning, […] the admission, you know how the kids are handed over to where they are going, a good communication, […]*	FG1
	*Participant: […] discharge planning for these kids, we know for maybe a week, or maybe not a week, but three or four days if they are going home, and we organize transport. […] plan everything for going home, […] then we have got all these obstacles which delays, then, the discharge*.	FG4
Learning from each other, sharing with new staff	*Participant: […] come out to the desk and be like lads, I've tried everything and the idea of getting a blood pressure cuff on or temps or whatever and then getting meds in. Then somebody will be like put it in a grape, he'll be like cool*.	FG1
	*Participant: […] one thing that I've seen quite a few people do, for example, if you are doing a set of obs or something, you can do it on yourself before you are doing it to them, to try and show that it's not harmful. […]*	FG5
Staying late to hand over all the important information	*Participant: When we have got multiple kids with disabilities on the ward, we will all be working back till later at night, […]*. *Participant: Sometimes you just have to […]. You've got to get your notes done. You've got to make sure that the patient's cared for*. *Participant: You need to make sure that you pass all the information on to the next nurse correctly, […] you need to go through their whole care regime, […], and then you are not leaving […]*.	FG4

### 4.5 Build trust and relationships

Nurses recognized the importance of building relationships with the CYP with intellectual disability, not just their parent, through the use of rapport. Sometimes the relationship development was opportunistic, but it was important to use the trust for continuity of care, to maintain and sustain the relationship if it works for the CYP with intellectual disability and their parent/carer. Often colleagues observed this and made note of the bond particular staff members have, identifying that these relationships were markers of good care experiences for CYP with intellectual disability.

Being empathic to build parental trust in the nursing staff was also seen as central to ensuring a good experience for the CYP with intellectual disability and their parent. Having a parent feel safe to leave their child's bedside overnight to get some rest was perceived by the nurses as an indicator of parental trust in nursing staff, and that they were doing a good job.

Building trust with the child and their family across the nursing team was also identified in one group; the staff worked together to give each child the best experience, acknowledging that some bonds are stronger than others and that the ward manager has an important role in advocating for the team and patients. Nurses in one group also wanted the organization to listen to them, and trust their expertise and knowledge of the needs of CYP with intellectual disability.

One group shared a story about a long term patient who the whole team get to know, and how as a team rallied together to give this child a good experience, to feel comfortable and safe. The team recognized that although the admission had been difficult, it was important that the child had a good experience of care. Key focus group extracts related to this theme are presented in Table 5.

**TABLE 5:** Build trust and relationships; key extracts from focus groups

Build trust and relationships	Key extracts from focus groups	Focus group reference
Continuity of care	*Participant: Today I had one of my patients, he'd been in bed all morning, […] one of the other nurses walked in, and his whole demeanour changed. […] he was so happy. He smiled and he was delightful, and he made some jokes. […]*	FG5
	*Participant: […] it's just finding the appropriate—the most appropriate person to interact with the children or with the parent. […] going with the person that they get along well with so that we can build a rapport with the individual ourselves […] then we are not reliant on the other people on after‐hours or weekends*.	FG6
Using empathy builds trust and parent feels safe to leave bedside	*Participant: […] when their kids are here quite regularly, […] they trust us. […] there will be parents who say he's in bed, asleep tonight. Like I know you'll ring me if there's any problems. I'll see you in the morning. Like that's nice to know that they will kind of relax a little bit and take the time to themselves […]*.	FG1
	*Participant: […] if I put myself into those parents' shoes, or that patient's shoes, to me, that's an expectation. I would expect it's part of your role to have that conversation with the mum and provide that care, not just go in and not acknowledge the child […]*.	FG5
Organization trusts nurses' clinical judgement	*Participant: If you provide feedback about a patient or we need this for patient A and B and they do not listen, and then maybe a week later, what we said initially is what happens. […]*	FG4
Build trust across the team with managers who advocate for the patients	*Participant: […] support from our manager in pushing for better staffing […] quite often, the parents will have other things that they need to do or have other kids that they need to look after. They cannot be expected to stay here with their kid […]*.	FG6
	*Participant: […] our managers are very good with building a relationship with the kids and the parents […]*.	FG6
Working as a team to ensure good experiences of hospital care	*Participant: […] we had a patient who was here for a long time, […] everyone had such a good relationship with him […]. It's not above and beyond, but it's more than what you would usually do. We would always come in and say hello, and give him a lot of attention, and he really thrived off that. […] You could see his eyes light up, […] those kind of little acts made a big difference for him*. *[…]* *Participant:[…] that little bit of extra effort to engage him with just some positive, fun, playful sort of banter,[…] it was yeah, a positive experience for him, even though it was a very difficult, long admission*.	FG5

### 4.6 Past experiences

The conceptual model refers to past hospital experiences for CYP with intellectual disability and their parent impacting on subsequent hospital experiences. For staff, the concept of learning from past experiences means they appreciate the need to plan for admissions, and, as in the second theme, know the child and their needs so the ward can prepare for the admission beforehand. Having knowledge of previous admissions and individual information documented in the medical record, particularly when the child had not been admitted to the ward before, was also helpful for optimizing the child's comfort during hospitalization.

For senior nurses, they also drew on their own past experiences of nursing CYP with intellectual disability to generate ideas for optimizing care and patient experience. They also identified that a good experience today for a CYP with intellectual disability can help reframe expectations of hospital and make for better future experiences. Being able to draw on past experiences also extended to optimizing patient safety by advocating for escalation of treatment and care, particularly in the context of a deteriorating CYP with intellectual disability.

Negative past experiences were also identified, and one group discussion revealed that having psychological support helped staff to cope with distressing events and return to work. Using past experiences to learn and improve, through parent and patient feedback was also identified. Key focus group extracts related to this theme are presented in Table 6.

**TABLE 6:** Past experiences; key extracts from focus groups

Past experiences	Key extracts from focus groups	Focus group reference
Use past documentation to plan for admission	*Participant: […] individualizing it to the patient, but also documenting it. […] you could look back and see what had worked in the past*.	FG5
Senior staff draw on their past experience	*Participant: […] Having all those conversations about their likes and dislikes and building rapport, […]. I started here probably about 10 years ago […] we had a little bit more time. […] to build rapport and consider their individual needs and to have time to respect their autonomy*.	FG2
	*Participant: I've worked here for quite a while and I've found a lot of strategies along the way of different ideas for things to try with different kids of different age groups and different abilities*.	FG3
Using past knowledge of CYP to advocate and escalate care	*Participant: Because if you know them quite well, you know when they are not acting like themselves and you can advocate for them. [….] A doctor that might not know them that well comes in and says, this is them and such is life, and you say, actually, we noticed X, Y, Z and that usually indicates that he's in pain or having a seizure. […] To advocate for them sometimes can be quite difficult […] the more senior staff that know the kids really well and their history advocate really well to make sure that we do not miss anything, even though they are so sick and so complicated*.	FG6
Support after a negative experience	*Participant: […] I had a day where I had a patient with an intellectual disability throw something across a desk towards us. […] that was quite distressing*. *[…] it was good that the hospital provided counselling the next day, […] that actually brought me back to work*.	FG4
Feedback from parents can help improve care delivery for the future	*Participant: […] training around encouraging parents to give that feedback whether it's good or bad so that we can make better changes in the future around the care that we give*.	FG3
	*Participant: When you save positive feedback from families or they comment on how well we work as a team and how happy their child is in our surroundings on ward*.	FG4

### 4.7 New themes

The analysis identified additional codes and themes across the transcripts that extended beyond the conceptual model; the unique role of a paediatric nurse, and joy and job satisfaction. These two themes are discussed below with a summary table of key extracts from the focus groups (Table 7). In addition, relevant to the context when we conducted the focus groups are the impacts of COVID‐19 pandemic restrictions. A summary table of key extracts for this theme are included in Table 8 below; this theme will be developed further and reported in a subsequent publication.

**TABLE 7:** New themes: Key extracts from focus groups

New theme	Key extracts from focus group	Focus group reference
The unique role of a paediatric nurse	*Participant: […] we are paediatric nurses, so we look after children across their lifespan. I guess from a communication perspective, we learn how to communicate whilst we are here, from the ages of a day old to an 18‐year‐old*.	FG5
	*Participant: […] trying to make time for […], a lot of us will just go in and say hi to the kid if they are actually in the room. […] even if it's not our patient, our own patient, if there's someone that their monitor is going off or we hear some strange noise coming from a patient's room, like coughing, we'll investigate, even though the patient might not be allocated to the specific nurse*.	FG6
Joy and job satisfaction	*Participant: And getting a smile. Getting a smile. […] Because half the time you go in and they are just looking at you like what are you going to do to me? Either petrified or angry. […] when you are going home and you are just like bye and they are like waving and smiling, you are just like oh okay. Back to work tomorrow, it's all right*.	FG1
	*Participant: […] we had one patient who was extremely difficult with a medical prognosis as well as an intellectual disability, […]. It was really lovely to be able to see him and us interact when things were going good for him. Things were not always so good. […] when we were having great days, it was nice to be able to interact with him*. *They would be singing songs, and we'd put videos on that he would enjoy, and then we'd be able to be part of that. Listening to him laughing, I think—I remember the first time I ever heard him laughing, and it was just such a joy to be able to hear him laugh in this particular time. It was a really sweet thing*. *Participant: I was there, and you…[…]…started crying*. *[…]* *Participant: I did. It was really lovely*.	FG4
	*Participant: I call parents […] I'm happy to call them every hour of the night if they want […]. I find when the parents are reassured, they—the child is also reassured, […] just communicating and always telling the child—even if you do not know how much they are absorbing but just what you are doing. I think that they just really appreciate it. You do get stuff back from them like smiles and laughter and all those really great things*.	FG6

**TABLE 8:** Impacts of COVID‐19: Key extracts from focus groups

New theme	Key extracts from focus group	Focus group reference
Impacts of COVID‐19 pandemic restrictions	*Participant: […] we have lost a lot of the resources on the ward […] we used to have a playroom on the ward that was full of toys and books and we do not have a playroom at all now. We do not have any toys on the ward. We used to have a whole toy cupboard. Then I think with the loss of the Child Life therapist as well all of that means we have a lot less resources […]. I know COVID has affected that as well but people were not able to […] do anything so I think we have just had a lot of kids stuck in bed with not much to do beyond an iPad which is not ideal*.	FG3
	*Participant: We had a child recently who was palliative. She came to us and was for end‐of‐life care. […] it was during the COVID crisis, and it was very distressing for the family. We were able to get the family down and to have them in our back dining room area, where we were allowed to have more people. […]. I really thought that was a really great initiative from the hospital and us, to be able to get her to actually have time with her family and see all of the people that love her and that she loves, and then she can—yeah, it was a really nice time. It was really lovely. […] that was really good thing that we have done*.	FG4

#### 4.7.1 The unique role of a Paediatric nurse

In each focus group discussion repeatedly referenced the role of the paediatric nurse. Participants consistently identified that the good aspects of care delivery were simply what they expected of themselves, how they identified with being a paediatric nurse. For all groups a central aspect of the role of a paediatric nurse was ensuring the children in their care have their time, with space and access to play while in hospital, and particularly for CYP with intellectual disability it was critical that accessible and appropriate means for play were available as well.

#### 4.7.2 Joy and job satisfaction

Associations between joy and job satisfaction were evident across the focus groups, often intertwined with the participants' descriptions of their role as a paediatric nurse. In practice, for the paediatric nurses in this study they described their interactions with CYP with intellectual disability as child‐centric, especially when talking about the moments which created joy and value in their work. Having time to just be with their patients, particularly to play and form connections, was seen as valuable and important for them in their role as a Paediatric nurse, but not considered critical for them to do their job as a nurse.

#### 4.7.3 Impacts of COVID‐19 pandemic restrictions

The focus groups were conducted during the COVID‐19 global pandemic. During this time the region where our study was conducted had not been under any significant restrictions for hospital visitors for several months. The focus groups presented a unique opportunity for the nurses to reflect on the impact of the restrictions in 2020 on their care delivery, and how they negotiated the restrictions safely to ensure good experiences of care for children and young people with intellectual disability. Key issues identified were a loss of resources, and finding ways to adapt care delivery within the restrictions.

### 4.8 Enhanced model of safe care for inpatient children and young people with intellectual disability

**FIGURE 3:**
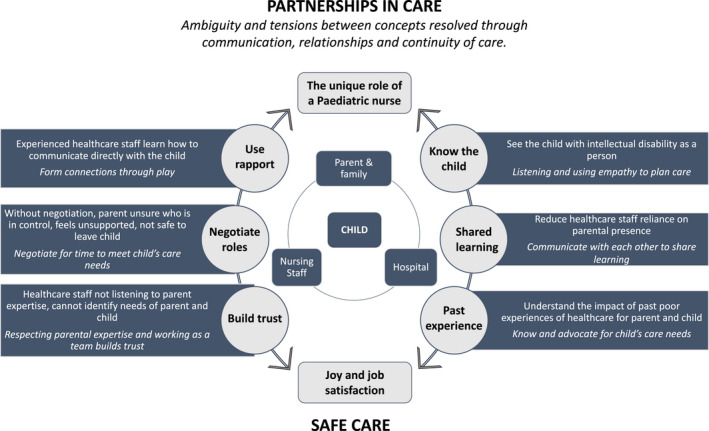
Enhanced model for safe care of inpatient children and young people with intellectual disability

The group discussions confirmed and identified possible extensions to the conceptual model of a good experience of care that creates safety for CYP with intellectual disability in hospital, in the context of inpatient tertiary paediatric healthcare specifically (see Figure 3). The enhanced model incorporates the perceptions of the paediatric nurses from our study and is extended to illustrate how the themes of the unique role of a paediatric nurse and joy and job satisfaction can represent positions of conflict and of harmony. Bringing in the perceptions of paediatric nurses confirmed the position of the child with intellectual disability being at the centre of safe care, where care is delivered as a partnership between nursing staff, parents/family and the hospital systems and processes.

In Box 1, we share a story from one group demonstrating how our enhanced model is applied in a single encounter that encapsulates the joy in paediatric nursing care experiences with a child with intellectual disability.

BOX 1 **Applying the enhanced model in a single encounter showing joy in paediatric nursing care**

A particularly touching story involved a senior nurse applying her expertise as a paediatric nurse, with joy and job satisfaction evident. The story depicts how this nurse uses play, reflects on her past professional and personal experiences, listens to the mother, uses rapport to learn about the child as a person and build trust, and shared learning, then applies these concepts in one beautiful yet humble description of taking a little bit of extra time and involving the child's favourite toy, a dinosaur:

*Participant: […] a little boy the other day who did not want to take his medication so I spent about 15 to 20 min finding ways to convince him to take it. Whereas before that mum had said, oh I sometimes just have to hold him down and squirt it in his mouth and then hold his nose shut which is traumatic for everyone involved. So then we tried to find different ways to motivate him to do it and in the end the thing that worked was getting him to be the fastest patient [in the] hospital*.

*So saying, oh, I've seen all these kids this morning take medication but I think you can be the fastest because he loved doing things quickly and he wanted to win the race. So we kind of worked a long way around to that and gave him an empty syringe to—so he could give his dinosaur some medication which he loved. Then I let him keep the syringe so he could playing with it. Then circled back to, oh, dinosaur took it fast. I think you could take it even faster than the dinosaur, and kind of convinced him that way. Mum was just really appreciative and happy and said she was going to try that at home when they were discharged rather than trying to just have this struggle day and night with medication*.

*Again, obviously took a lot longer than the 10 s to hold him—pin him down and squirt it in but it was really worth it and mum was really appreciative and the little boy was happy that he'd given his dinosaur some medication and had taken his as the fastest kid in the hospital too. Yeah, I think the length of time in coming up with different strategies to really take the time to give good care rather than rushing around and feeling like you cannot spend that time with patients. Just listening to parents and carers so I think—I've been to hospital with my own kids and when people do not listen to you it's incredibly frustrating. So I think for me, listening to the parents and carers and taking on board what they are saying and trying to make a difference, yeah, is part of good hospital care*.

## 5 DISCUSSION

Nurses in this study described good experiences of care for CYP with intellectual disability in hospital as exemplified by working in partnership with parents to understand their child's needs and adapting care delivery to optimize quality and safety and enhance the patient experience. This involved forming connections, effective communication to develop relationships and continuity of care to maintain trust. Using rapport through play to get to know the child with intellectual disability, listening to and learning from expertise of parents and their colleagues, and using empathy to advocate for their patient and family, sometimes working past their allocated shift hours, were all identified by the nurses as ‘just what we do’ to make the best of each hospital experience for CYP with intellectual disability. Using a positive deviance lens elicited experiences of the nurses in our study that complement our conceptual model of safe care, demonstrating how the key elements of the model have practical applications for the clinical setting.

Using play to form relationships was repeatedly mentioned by the nurses as central to good nursing care of hospitalized children with intellectual disability. In particular the effort taken by some of the nurses to find ways to form connections with a child, especially those who do not communicate verbally, seeking opportunity to connect by finding out how the child communicates, showing empathy and compassion. In several groups this led to some discussion on what it means to be a paediatric nurse, and the importance of Child Life Therapists for children in hospital, especially CYP with intellectual disability. The use of play to support the child to build resilience, develop effective coping strategies, and a positive hospital experience are key facets of the Child Life Therapist role (Humphreys & LeBlanc, 2016). However, the nurses also identified the importance of having time to play with their patients particularly CYP with intellectual disability, which should be acknowledged as a key component of their role as a paediatric nurse.

Across all groups the nurses identified that getting to know and provide safe care for a CYP with intellectual disability takes time and this raised conflicting role priorities between the organizational expectations and nurse's beliefs. This has been identified in other studies (Ford & Turner, 2001; Lewis et al., 2019). Ford and Turner (2001), found nurses discussed the guilt of knowing the parental expectations for their hospitalized child, and wanting to meet these expectations, creating tensions when the nurses could not find the time to give the care they believed the child deserved. Conversely, organizational consideration of paediatric nurses perceptions of the needs of their patients when managing day to day ward needs may impact on care quality. A recent scoping review of burnout in paediatric nurses found feelings of accomplishment in paediatric nurses was associated with mother satisfaction with meeting child's care needs (Buckley et al., 2020). Furthermore, Oulton et al. (2015) found that staff taking time to find out the ‘little things’, particularly non‐medical needs, about the CYP with intellectual disability may optimize the quality and safety experience of hospital.

In some groups partnerships with parents were discussed in the context of family centred care (FCC), as a framework for their practice though many of the stories from the nurses in our study were primarily child‐centric. FCC is described as care that is planned and coordinated around the needs of the family rather than the child/person (Coyne, 2015; Jolley & Shields, 2009; Watts et al., 2014). Despite the popularity of FCC, evidence of its effective implementation for optimal paediatric healthcare remains inconclusive, particularly where the principles are misunderstood (Coyne, 2015; Kuo et al., 2012; Watts et al., 2014). A critical challenge to FCC is the focus of healthcare shifts away from the child, to the parent and family (Mattsson et al., 2013). For example, while most nurses in our study agreed role negotiation, shared care and partnerships were part of effective FCC, in one group the nurses flagged an expectation for the parent to be at the bedside for FCC to occur. Conflicting use of FCC is in line with other studies exploring hospital care from the perspective of paediatric nurses and parents of CYP with intellectual disability (Aston et al., 2014a, 2014b; Oulton et al., 2015). The enhancements to our conceptual model confirm the underlying nursing focus; with the child is in the centre of a care partnership, the child's individual needs are at the centre of the care experience.

Our findings repeatedly demonstrate that experienced nursing staff recognize the importance of past experience(s), including pre‐registration training and education, and clinical exposure to build care partnerships to optimize the delivery of high quality care of hospitalized CYP with intellectual disability, and this is readily discussed across the literature (Breau et al., 2018; Lewis et al., 2019; Oulton et al., 2015). Furthermore, parental healthcare seeking behaviour, particularly their choice of health services for their CYP with intellectual disability may be influenced by past experiences and relationships with health professionals. Parents of CYP with intellectual disability value and will utilize services where healthcare staff value relationships, partnerships and continuity of care with the parent and child, and have knowledge of their child's disability (Fereday et al., 2010; Smith et al., 2015). The lack of experience, knowledge and training on the part of health professionals contributes to poor experiences (Iacono et al., 2014). By incorporating the practice based knowledge of nurses employed in children's hospitals into our conceptual model we illustrate how the nurse‐parent–child partnership is developed in practice, when caring for CYP with intellectual disability in a tertiary children's hospital.

A key strength of our study is using qualitative inquiry with a positive deviance approach to explore paediatric nurse perceptions of good experiences of care. As the nurses in this study noted, paediatric nurses do not often take opportunity to reflect on the good things in their work; the nurses consider their interactions with children and parents as ‘just what we [paediatric nurses] do’. Furthermore, it was the supportive dynamic created through the focus group format that enabled the sharing and co‐construction of the nurses' stories of good care experiences that gave us the rich data in this study (Kitzinger, 1994). Perhaps one of the most delightful findings from the positive deviance approach using of focus groups were the feelings of joy that the paediatric nurses in this study expressed about their past experiences caring for CYP with intellectual disability; the nurses consistently talked of seeing ‘smiles’, the laughter, tears of joy for the smallest achievements of the children in their care. The authors found the focus group discussion to be especially valuable for revealing these joys.

### 5.1 Limitations

The participants in this study were all nurses from a single paediatric healthcare organization with two tertiary children's hospitals in the same metropolitan city. While this was intended, the experiences for paediatric nurses working in children's wards situated in adult tertiary or district hospitals, or experienced paediatric nurses who are not frequently exposed to CYP with intellectual disability, may have difference perspectives that were not captured here. In addition, our study was conducted during COVID‐19 pandemic and this may have influenced the stories and experiences of the participants. However, we believe this context also produced some of the rich stories, as the nurses were able to reflect and describe how they adapted care for CYP with intellectual disability when their usual resources, such as play rooms, toys and ward volunteers were limited.

As a research team, we were looking for themes that would align with our conceptual model; focusing on this aim during our analysis risked that we would miss other important concepts, negative cases or orphan themes. This was managed by using a two phased approach to the analysis, IPA and then Framework analysis. In this way we were able to identify important concepts and themes that sat outside the model. We then used the Framework method to map themes to the conceptual model and use the new themes to enhance the model for clinical application.

### 5.2 Recommendations for practice

The model presents a practical guide for new graduate nurses, or those with minimal experience caring for CYP with intellectual disability. For nurse managers, health service managers and policy makers, the model may serve as a framework for assessing and advocating for the day to day needs of their wards, staff and patients, and when developing and revising policies and procedures to guide staff in the delivery of safe care for CYP with intellectual disability in hospital. Finally, the enhanced model poses an opportunity for health services to look beyond current indicators of safe, quality care and look to using the model as a basis for patient experiences of care partnerships for inpatient CYP with intellectual disability.

## 6 CONCLUSION

This study used focus groups to explore perceptions of care experiences of care from paediatric nurses with experience caring for CYP with intellectual disability in hospital. The findings highlight the importance of developing partnerships with parents for safe care, and the significance of using play to develop rapport and taking time to become familiar with the needs of the CYP to optimize the quality and safety of hospital care. We have used the findings from this study to elaborate and enhance our conceptual model of safe care for CYP with intellectual disability, centred on the child and their care experience with a focus on quality and safety. Enhancements to the model reflect the unique role of a paediatric nurse in providing specialized inpatient care for CYP with intellectual disability, and the joy and job satisfaction this specialized role brings.

## CONFLICT OF INTEREST

No conflict of interest has been declared by the authors.

### PEER REVIEW

The peer review history for this article is available at https://publons.com/publon/10.1111/jan.15256.

## Supporting information

Supinfo

Appendix

## Data Availability

The data that support the findings of this study are available from the corresponding author upon reasonable request.
