# Supplementary material for: ‘Smiles and laughter and all those really great things’: Nurses' perceptions of good experiences of care for inpatient children and young people with intellectual disability
Source: J Adv Nurs. 2022 Apr 22;78(9):2933–48. doi: 10.1111/jan.15256 (PMC9544709; doi:10.1111/jan.15256)
Supplement: Supplementary file 2 — Appendix [file JAN-78-2933-s002.docx]

Appendix 1

Focus group questions – ward staff

Version 2.0

15^th^ April 2020

Alternative conduct of focus groups if Public Health order remains in place:

Skype or video conference (zoom) either from home or utilising various computers within the ward/department (will liaise with NUM/head of dept)

Introduction:

Hello everyone and thank you for coming today.

My name is [external facilitator to explain who they are and their role as facilitator]

The purpose of this focus group is…

Can I check that everyone has received an information sheet and given a signed the participant consent form? Just reaffirming now that participation is voluntary and you are free to leave at any time.

Guidelines for focus groups:

Thinking about the last time you cared for a child with intellectual disability on this ward:

1. What aspects of hospital make for a good and safe experience of care for a child with intellectual disability?

- What do you think you, as individuals do well?
- What do you think is done well as a ward or unit, a hospital?

1. What aspects of hospital make for a bad and unsafe experience of care for a child with intellectual disability?
2. What factors (patient, staff, organisation) facilitate or hinder a good experience of hospital for a child with intellectual disability?
